# Correction: Tang et al. Integrated Multi-Omics Analysis Reveals Mountain-Cultivated Ginseng Ameliorates Cold-Stimulated Steroid-Resistant Asthma by Regulating Interactions among Microbiota, Genes, and Metabolites. *Int. J. Mol. Sci.* 2024, *25*, 9110

**DOI:** 10.3390/ijms251810021

**Published:** 2024-09-18

**Authors:** Daohao Tang, Chao Wang, Hanlin Liu, Junzhe Wu, Luying Tan, Sihan Liu, Haoming Lv, Cuizhu Wang, Fang Wang, Jinping Liu

**Affiliations:** 1 School of Pharmaceutical Sciences, Jilin University, Changchun 130021, China; tangdh22@mails.jlu.edu.cn (D.T.); hanji23@mails.jlu.edu.cn (H.L.); wujz23@mails.jlu.edu.cn (J.W.); tanly23@mails.jlu.edu.cn (L.T.); lvhm2822@mails.jlu.edu.cn (H.L.); wangcuizhu@jlu.edu.cn (C.W.); 2 College of Basic Medical Sciences, Jilin University, Changchun 130021, China; wangc19@mails.jlu.edu.cn; 3 College of Animal Science and Technology, Jilin Agricultural University, Changchun 130118, China; liusihan040306@126.com

In the original publication [1], reference 16 was incorrectly cited. We have deleted the old reference 16 [2] and added the corrected reference [3] instead.

The authors state that the scientific conclusions are unaffected. This correction was approved by the Academic Editor. The original publication has also been updated.
