# Supplementary material for: Integrated Multi-Omics Analysis Reveals Mountain-Cultivated Ginseng Ameliorates Cold-Stimulated Steroid-Resistant Asthma by Regulating Interactions among Microbiota, Genes, and Metabolites
Source: Int J Mol Sci. 2024 Aug 22;25(16):9110. doi: 10.3390/ijms25169110 (PMC11354367; doi:10.3390/ijms25169110)
Supplement: Supplementary file 1 [file ijms-25-09110-s001.zip › ijms-3166950-supplementary.pdf]

Table S1 RT-PCR primer information

Primer Information	Primer name	Primer name(5'-3')	Clip length (bp)	Annealing temperature(°C)
NM_008084.2	M-GAPDH-S	CCTCGTCCCGTAGACAAAATG	133	60
	M-GAPDH-A	TGAGGTCAATGAAGGGGTCGT		60
NM_009386.2	M-ZO1-S	GGGAAAACCCGAAACTGATG	103	60
	M-ZO1-A	GCTGTACTGTGAGGGCAACG		60
NM_001360536.1	M-Ocln-S	TCTGCTTCATCGCTTCCTTAGT	189	60
OCCLUDIN	M-Ocln-A	AGCCGTACATAGATCCAGAAGC		60
NM_016674.4	M-claudin 1（5）-S	GTGTCCTACTTTCCTGCTCCTGT	101	60
	M-claudin 1（5）-A	TCACACATAGTCTTTCCCACTAGAAG		60
NM_010844.3	M-MUC5AC-S	GAGCCTTCAACCCAGGACATAC	266	60
	M-MUC5AC-A	CGGGTGGTTATTGACTAAGACG		60

Labeling of upstream primers：-S(sense primer)

Labeling of downstream primers：-A(antisense primer)

Section S1 Chromatographic and mass spectrometric conditions for the detection of SCFAs

Gas chromatography conditions

The GC analysis was performed on trace 1300 gas chromatograph (Thermo Fisher Scientific, USA). The GC was fitted with a capillary column Agilent HP-INNOWAX (30 m × 0.25 mm ID × 0.25 μm) and helium was used as the carrier gas at 1 mL/min. Injection was made in split mode at 10:1 with an injection volume of 1 μL and an injector temperature of 250°C. The temperature of the ion source and MS transfer line were 300°C and 250°C, respectively. The column temperature was programmed to increase from an initial temperature of 90°C, followed by an increase to 120°C at 10°C/min, and to 150°C at 5°C/min, and finally to 250°C at 25°C/min which was maintained for 2 min .

Mass spectrum conditions

Mass spectrometric detection of metabolites was performed on ISQ 7000 (Thermo Fisher Scientific, USA) with electron impact ionization mode. Single ion monitoring (SIM) mode was used with the electron energy of 70 eV.

Section S2 Chromatographic and mass spectrometric conditions for untargeted metabolomics

Mass spectrum conditions

Waters Xevo G2-XS QTOF high resolution mass spectrometer can collect primary and secondary mass spectrometry data in MSe mode under the control of the acquisition software (MassLynx V4.2, Waters). In each data acquisition cycle, dual-channel data acquisition can be performed on both low collision energy and high collision energy at the same time. The low collision energy is 2V, the high collision energy range is 10~40V, and the scanning frequency is 0.2 seconds for a mass spectrum. The parameters of the ESI ion source are as follows: Capillary voltage: 2000V (positive ion mode) or -1500V (negative ion mode); cone voltage: 30V; ion source temperature: 150°C; desolvent gas temperature 500°C; backflush gas flow rate: 50L/ h; Desolventizing gas flow rate: 800L/h.
